# The gendered costs of human–wildlife conflict: A global systematic review

**DOI:** 10.1007/s13280-025-02300-y

**Published:** 2025-11-07

**Authors:** Katie A. Adler, Meredith L. Gore, Christine E. Wilkinson

**Affiliations:** 1 Department of Ecology and Evolutionary Biology, University of California, Los Angeles, Los Angeles, CA 90024 USA; 2 Department of Geographical Sciences, University of Maryland, College Park, MD 20740 USA; 3 Department of Community Science, Natural History Museum of Los Angeles County, Los Angeles, CA 90007 USA; 4 California Academy of Sciences, San Francisco, CA 94118 USA

**Keywords:** Economic costs, Gendered costs, Hidden damages, Human–wildlife interactions, Physical costs, Social costs

## Abstract

Human–wildlife conflict is a pressing worldwide issue with consequences that are experienced differently across groups of people. Societal expectations and gender norms ensure that the consequences of these conflicts have nuanced, gender-dependent implications. We conducted a systematic literature review on human–wildlife conflict to synthesize gendered costs. We examined six cost types: economic, physical, psychological, social, conservation management participation, and wildlife crime, and found that these costs varied significantly by gender. We also found that costs have gendered consequences for how people perceive the wildlife involved in these conflicts. We identify a range of opportunities and entry points for managers, researchers, and policymakers interested in reducing costs associated with human–wildlife conflict by more directly accounting for gender.

**Supplementary Information:**

The online version contains supplementary material available at 10.1007/s13280-025-02300-y.

## Introduction

Human–wildlife conflicts (HWCs)—such as livestock predation, wildlife trafficking, crop raiding, attacks on people, and retaliation against wildlife—are present across societal and ecological contexts and present a global challenge. Ongoing climate change and urbanization are exacerbating the frequency and severity of HWC via increased human–wildlife overlap, range shifts, altered human behaviors, and resource competition (Abrahms et al. 2023; Ma et al. 2024). For example, intensified droughts have exacerbated crop raiding by African elephants (*Loxodonta africana*) (Mariki et al. 2015), changes in forage availability for wild prey have led to range shifts for snow leopards (*Panthera uncia*) that fuel increased predation on livestock (Aryal et al. 2014a, 2014b), and urban sprawl has made conflict-influencing anthropogenic attractants widely available for wildlife (e.g., (Soulsbury and White 2015; Raymond and St. Clair 2023). Along with these realized HWCs, people’s risk perceptions toward wildlife can fuel retaliation against species involved in conflicts and influence whether they will or will not support conservation measures (Gore et al. 2008; Dickman 2010; Gore and Kahler 2012; Wilkinson et al. 2021).

The impacts of HWCs are unevenly distributed across communities (Braczkowski et al. 2023; Harris et al. 2023), disproportionately affecting certain groups based on multiple factors including livelihood, socioeconomic status, and cultural context. For example, pastoralists and small-scale farmers are typically more vulnerable to losses from livestock predation by large carnivores or crop raiding by ungulates. Further, subsistence lifestyles and the size of their herd or farm can typically make it more difficult for someone to cope with or recover from the impacts of those conflicts (Braczkowski et al. 2023) and communities living subsistence lifestyles alongside protected areas may be particularly vulnerable to wildlife-borne costs while seeing little benefit (Jordan et al. 2020). The impacts of HWC may also intersect with who holds decision-making authority (Lute and Gore 2014; Lute et al. 2016), such as who controls access to mitigation tools or who is eligible for compensation (Hamm et al. 2024). This can result in unequal ability to respond to or prevent future incidents.

Gender is one critical lens through which to understand the inequities inherent to HWC impacts, but it remains understudied (Gore and Kahler 2012). Gender roles often dictate who performs tasks, such as collecting water or guarding crops, and thereby shape patterns of exposure and vulnerability to HWC. For example, women may face elevated risks while conducting subsistence activities like firewood collection, which may bring them in frequent proximity to wildlife (Ogra 2008). Gender roles can influence decision-making processes, access to resources, and participation in conservation planning (James et al. 2021). Furthermore, there may be gender-specific indirect or hidden costs from HWC, such as increased labor burdens, stigma, and mental health consequences (e.g., Doubleday and Adams 2020). These impacts can be shaped not only by gendered divisions of labor, but also by broader societal norms, religious or cultural taboos, and governance structures that may limit the involvement of women and other marginalized genders in conflict management (Ogra 2008).

Despite growing recognition of the importance of equity in conservation (e.g., Friedman et al. 2018; Law et al. 2018; Davis 2019), the gendered dimensions of HWC remain poorly understood. Understanding how gender intersects with HWC and how costs are borne across genders will be key for holistically and equitably addressing HWC, especially as we grapple with ongoing global change. To help address this gap and inform more inclusive approaches to coexistence, we conduct a global systematic review of the gendered dimensions of HWC. Specifically, we (1) curate a novel global dataset about literature on gender and HWC, (2) review and synthesize the content of this dataset, (3) identify patterns, trends, and gaps in knowledge regarding gendered costs of HWC, and (4) provide a framework for integrating gender into HWC research, prevention, and mitigation.

## Methods

### Search methods

To synthesize the gendered costs of human–wildlife conflicts, we followed the guidelines reported by Collaboration for Environmental Evidence (2018) to conduct a systematic literature search. Within the Web of Science (WOS) Core Collection, we used the following keywords: TS = (*human wildlife conflict* AND gender*) OR TS = (*human–wildlife conflict* AND gender*) OR TS = (*human–wildlife conflict* AND *feminist political ecology* AND *gender*) OR TS = (*hidden cost* AND *gender* AND *human–wildlife conflict*) OR TS = (*human–wildlife conflict* AND gendered vulnerabilit*) OR TS = (*human-carnivore conflict* AND *gender*) OR TS = (*human-primate conflict* AND *gender*) OR TS = (*human-elephant conflict* AND *gender*) OR TS = (*human–wildlife conflict* AND *women*) OR TS = (*human–wildlife conflict* AND *women*) OR TS = (*human–wildlife interactions* AND *gender*) OR TS = (*human wildlife interactions* AND *gender*) OR TS = (*human-canid conflict* AND *gender*) OR TS = (*human–wildlife conflict* AND feminis*) OR TS = (*human-bear conflict* AND *gender*) OR TS = (*wildlife crime* AND *gender*) OR TS = (wildlife traffick* AND *gender*) OR TS = (*wildmeat trade* AND *gender*) OR TS = (*bushmeat* AND *gender*) OR TS = (*wildmeat* AND *gender*) OR TS = (zoono* AND *wildlife* AND *gender*) OR TS = (*disease* AND *wildlife* AND *gender* AND human*) OR TS = (conservation conflict* AND *wildlife* AND *gender*) OR TS = (poach* AND *wildlife* AND *gender*). Our final search was conducted on June 4, 2024 and produced 324 papers published between 1998 and 2024. Comprehensive PRISMA diagrams of our screening process (see below) can be found in Figs. 10.1007/s13280-025-02300-y–10.1007/s13280-025-02300-y.

### Exclusion criteria and screening process

Following the PRISMA 2020 guidelines (Page et al. 2021), we created exclusion criteria to determine which papers to include in our review. We only included papers accessible in the English language that discussed vertebrate animals and human gender and required that included papers had a focus on human–wildlife interactions and how human gender has a role in those interactions. Papers focused on zoonotic disease within humans or livestock were only included if they discussed gendered costs of transmission; we excluded papers focused on the microbiological mechanisms of zoonotic transmission (bacteria and/or viruses). We only included papers focused on the perceptions of human–wildlife conflict as related to gender if they discussed the reasoning behind why those perceptions arise. All books, book chapters, and review papers were excluded, as well as gray literature.

Upon determining our exclusion criteria, we performed initial screening—again following the PRISMA 2020 guidelines—by title and abstract, followed by full text screening of each remaining publication (Page et al. 2021). In addition, using information gathered during both the abstract and full text screening, we conducted forward–backward searching for any additional papers that we may have missed during our initial WOS literature search. To do so, we re-read papers that we included in the final screening process of our initial search and created a list of any relevant papers that were referenced in these studies. We then performed the screening process on these additional publications. After screening, our final dataset consisted of 121 publications (see link for dataset under Data Accessibility).

### Data extraction

After finalizing our list of included publications, we categorized each publication based on geographic locations, topic, methods used, and cost types discussed. We identified the continent and country where each study was focused, as well as whether the study took place in multiple countries. Methodology was categorized as quantitative, qualitative, or both, and we noted whether the methods involved conducting an interview or survey. Each publication was classified by what was being measured regarding human–wildlife conflict: perceptions of wildlife, perceptions of hidden costs, knowledge and/or perceptions of zoonoses, wildlife-related mortality, wildlife crime involvement, involvement in hunting and/or wild meat consumption, wildlife presence and destruction, and perceptions of wildlife versus assessed wildlife presence (Table 1). We noted whether gender was the central focus of the paper and whether there was a specific focus on a particular gender. If a paper asserted that, within the context of their study, people of a specific gender were experiencing more overall costs than those of other genders, the affected gender was noted. Any other central focus of each paper and any benefits that people of specific genders may have experienced because of human–wildlife conflict were noted. Finally, we recorded the wildlife species or taxa that was involved in the discussed human–wildlife conflict.

**Table 1:** The percent of total studies (*n* = 121) in our dataset encompassing each measure examined in the context of human–wildlife conflict

Measure	% Total studies
Perceptions of wildlife	47.5
Perceptions of hidden costs	17.2
Other—*Perceptions of conservation management; Perceptions of poaching; Roles in zoonotic disease transmission*	10.7
Knowledge and/or perceptions of zoonoses	7.38
Wildlife-related mortality	6.56
Wildlife crime involvement	6.56
Wildlife presence and destruction	5.74
Involvement in hunting and/or wild meat consumption	4.10
Perceptions of wildlife versus actual wildlife presence	0.82

A ‘cost’ was defined as a negative outcome for one or more persons that is directly or indirectly caused by an interaction with wildlife. While there are certainly other hidden costs, we determined that there are six major cost types represented in the literature: conservation management, economic, physical, psychological, social, and wildlife crime. We recorded the type of cost experienced in each paper, as well as specific subtypes of costs. Conservation management costs were related to exclusion from conservation activities and planning in relation to HWC. Economic costs related to direct or indirect detrimental fiscal losses due to conflicts with wildlife. Physical costs involved tangible harm or violence toward one or more people as a direct or indirect result of HWC. Psychological costs were related to the internalized mental health consequences that conflicts with wildlife may create. Social costs were related to the responses of those who may be peripherally affected by HWC and the perception changes that may result from a conflict with wildlife. Wildlife crime costs related to differential participation in wildlife trafficking and poaching activities and their consequences. We recorded explanations the authors gave for results related to gendered costs of human–wildlife conflict. If results related to people’s gendered perceptions of the costs associated with human–wildlife conflict, we noted whether the reasoning for these perceptions given by the authors was speculative. In other words, we documented whether the authors based their discussion regarding perceptions of HWC-related costs on past literature or whether they directly asked the interviewees to provide insights on why they felt these perceptions held true in their communities.

### Statistical analysis

All statistical analyses and data visualizations were conducted using R (version 4.4.1; R Core Team 2024). To identify significant patterns and trends in gendered costs, we conducted Fisher tests using the ‘stats’ package (R Core Team 2024) since our data did not meet assumptions of normality. We analyzed the following comparisons: cost type by gender, physical costs by gender, psychological costs by gender, cost type by continent, social costs by continent, physical costs by continent, economic costs by continent, wildlife crime costs by continent, and psychological costs by continent. We did not run a Fisher test for economic costs by gender, social costs by gender, wildlife crime costs by gender, conservation management costs by gender, or conservation management costs by continent because of limited sample sizes for these costs across different genders or across cost subcategory (specifically for conservation management by continent), as most subcategories had zero studies. We created plots using the ‘ggalluvial’ (Villanueva and Chen 2019) and ‘ggplot2’ (Villanueva and Chen 2019) packages.

## Results

Our final dataset (Adler et al. 2025) consisted of 121 peer-reviewed publications from 1987 to 2024, providing the most comprehensive list of publications focused on the gendered costs of human–wildlife conflict to date. Although we included publications from as early as 1987, 98% were published during or after 2000, suggesting that the study of human–wildlife conflict’s consequences across genders is new. Most publications (*n* = 84) were not directly focused on the gendered costs of HWC. Publications that did not focus on a specific gender comprised most of our dataset; only sixteen studies focused on a specific gender. Notably, all sixteen studies focused on women. Across the entire dataset, we did not find any studies focused on genders other than the binary man or woman.

Most studies contained a mix of quantitative and qualitative methods (*n* = 81), followed by quantitative only (*n* = 25) and qualitative only (*n* = 15). Data collection methods employed by authors to achieve research objectives included having study participants draw maps to represent instances of carnivore conflict across their community (e.g., Wilkinson et al. 2021) and using a board game to examine differences across conflict perceptions (e.g., Sargent et al. 2022). Other studies utilized wildlife trafficking and court records (e.g., Sollund 2020), social media (e.g., Mkono et al. 2021), and livestock compensation records (e.g., DeMotts and Hoon 2012).

### Geographic trends

**Fig. 1:**
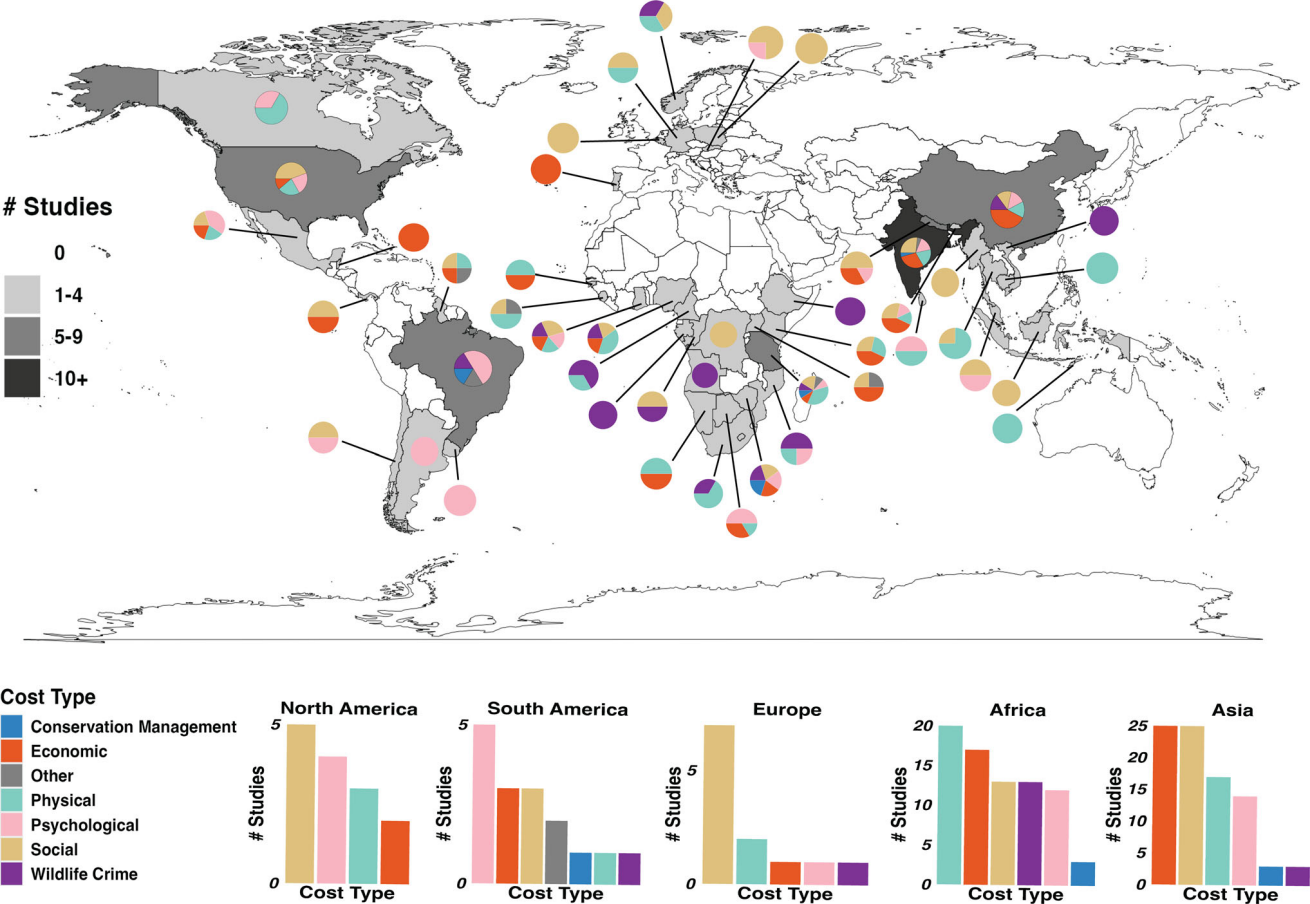
A global map of the number of reviewed studies per country. Bar charts depict the number of studies for each gendered cost type by continent, and pie charts depict the proportion of studies for each country covering each gendered cost type

**Fig. 2:**
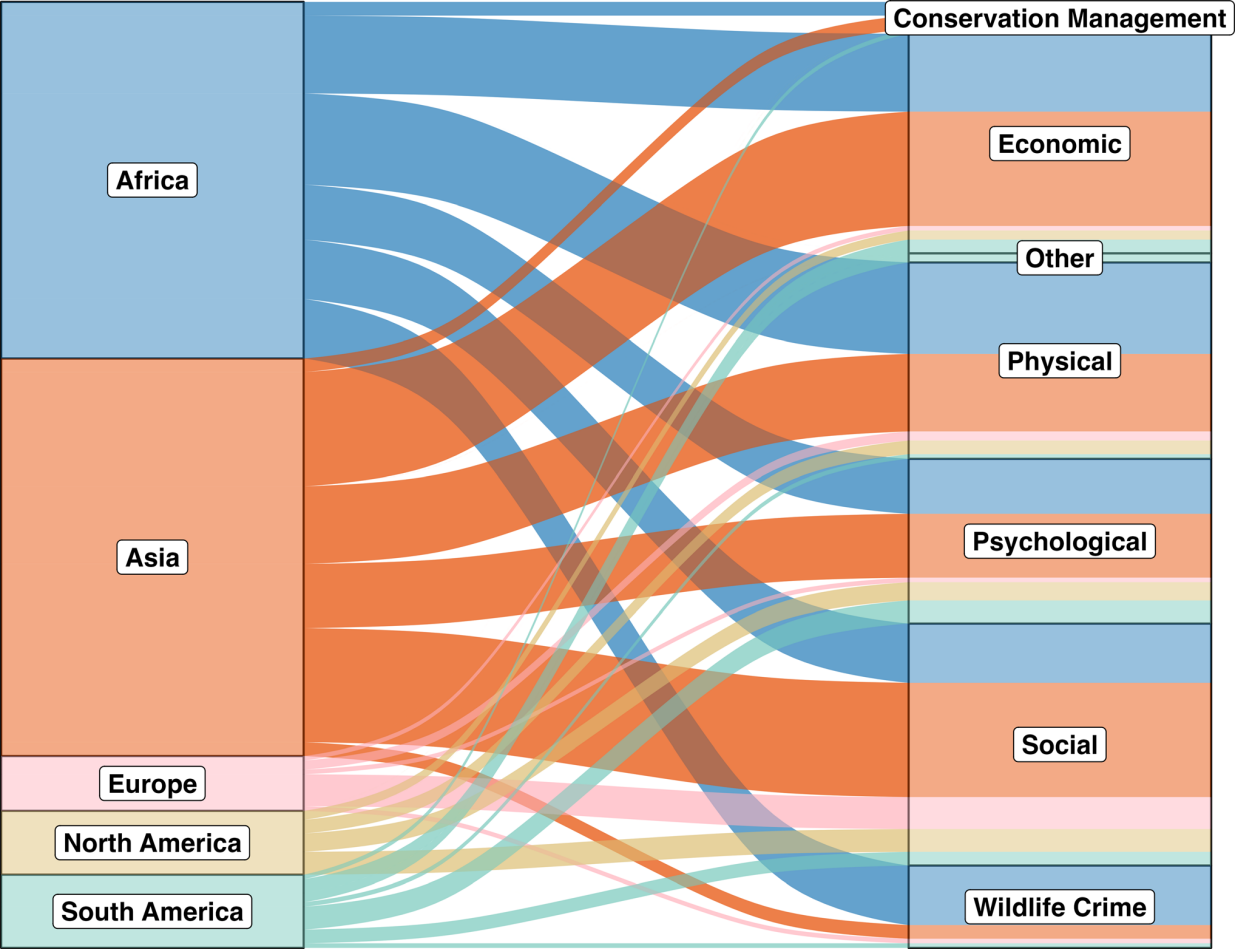
Human–wildlife conflict costs across continents, as cited in reviewed literature (*n* = 121 publications) that focused on gendered costs of human–wildlife conflict

We found that studies were geographically skewed (Fig. 1) but that costs did not vary significantly across continents (*p* = 0.08; Fig. 2). Forty-two studies were conducted in Africa, 47 were conducted in Asia, nine were conducted in Europe, and eleven were conducted in North America and South America each. At the regional level, 23 studies were conducted in South Asia, 20 of which were in India, followed by 14 in East Africa, seven of which were in Tanzania.

### Taxonomic trends

Thirty-five species and five different clades were reported to be involved in HWC. Elephants (ten publications; multiple species) and tigers (nine publications; *Panthera tigris*) were the species most discussed across the studies. Ten publications were related to conflict with carnivores, as compared to 75 papers that discussed conflict with other species. All other papers discussed conflict with wildlife broadly and were not related to a specific species.

### Distribution of cost types

**Fig. 3:**
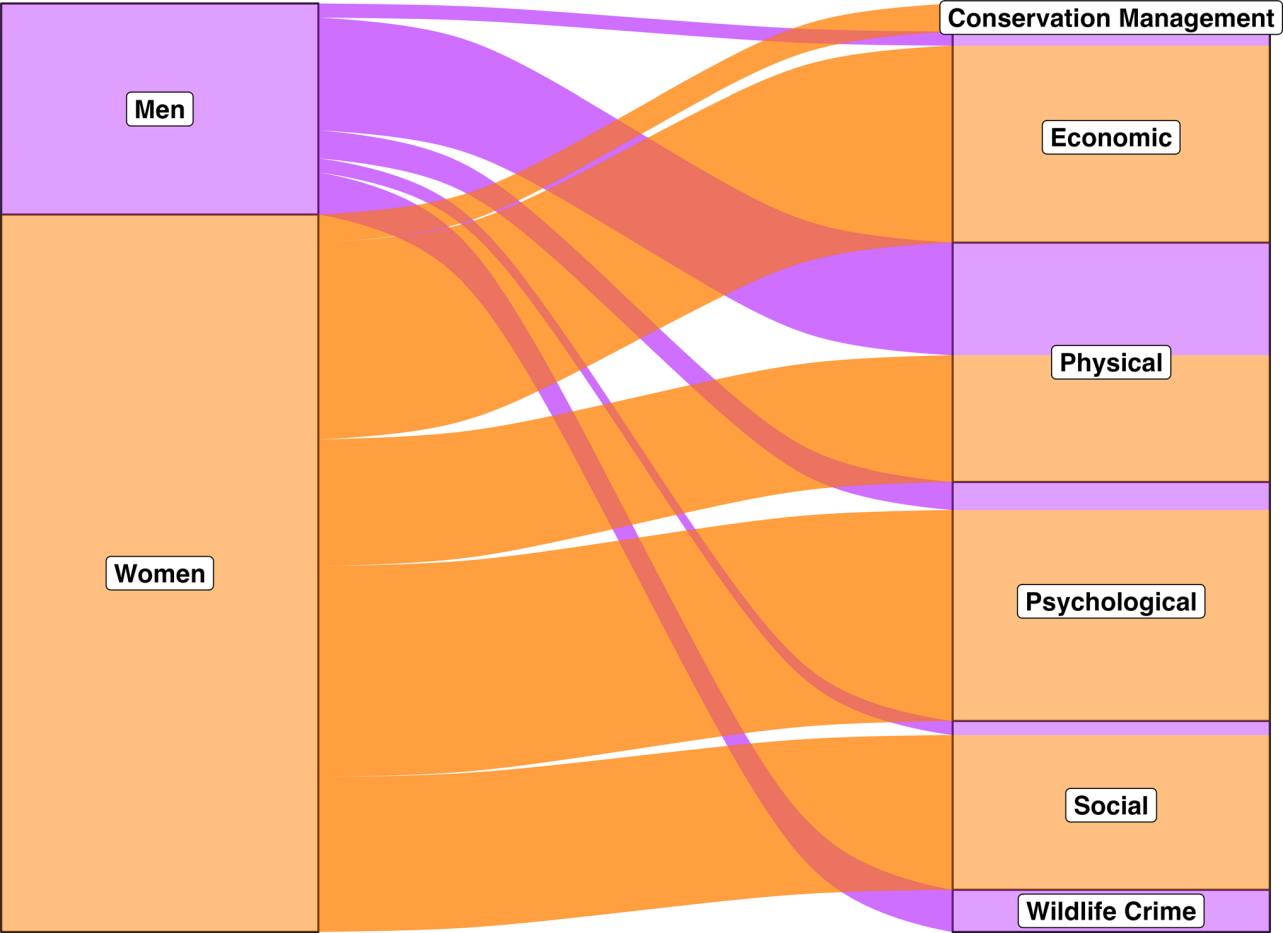
Human–wildlife conflict costs across genders, as cited in reviewed literature (*n* = 121 publications). Note that none of the reviewed literature assessed genders outside of the man/woman binary

Costs varied significantly across genders (*p* = 0.001; Fig. 3), but we did not find variation across continents for each cost (social: *p* = 0.9; physical: *p* = 0.5; economic: *p* = 0.6; wildlife crime: *p* = 1.0; psychological: *p* = 1.0; conservation management: *p* = 1.0). Physical costs varied significantly by gender (*p* = 0.005) while psychological costs did not (*p* = 0.2). Sixty-eight papers discussed economic costs, 56 papers discussed social costs, and 43 papers discussed psychological costs (Table 2). Physical costs were represented the most, with 75 papers discussing at least one subcategory of physical costs (Table 2). We recorded 21 papers that discussed wildlife crime and only four papers on participation in conservation management (Table 2).

**Table 2:** The percent of studies within each broad cost type that discuss each cost subcategory

Cost type	% Studies within each cost type
*Economic cost* (*n* = 68)	
Loss of food source	52.9
Loss of income source	32.4
Loss of property	13.2
Other—*Lack of financial independence*	1.47
*Physical cost* (*n* = 75)	
Fatal injury	20.0
Nonfatal injury	20.0
Zoonotic transmission to humans	20.0
Zoonotic transmission to domesticated animals	10.7
Decreased personal care	8.00
Increased workload	6.67
Violence toward person	6.67
Defense of property	4.00
Violence toward wildlife	2.67
Defense of self and/or others	1.33
*Psychological cost* (*n* = 43)	
Fear of wildlife-related injury or death of human	51.2
Mental health	20.9
Other—*Lack of confidence; Zoonoses knowledge*	9.30
Fear of wildlife-related injury or death of livestock	6.98
General fear and/or antipathy toward wildlife	6.98
*Social cost* (*n* = 56)	
Perceptions of conflict	75.0
Other—*Wildlife values; Perceptions of conservation management & management areas; Gendered conservation management initiatives*	8.93
Culture	7.14
Stigma	7.14
Religion	1.79
*Wildlife crime cost* (*n* = 21)	
Crime involvement as offender	57.1
Crime involvement as intermediary	19.0
Crime involvement as recipient of illegal item(s)	14.3
Perceptions of wildlife crime	9.52
*Conservation management cost* (*n* = 4)	
Conservation exclusion	75
Conservation inclusion	25

Economic costs (*n* = 68) consisted of 36 studies discussing losses of food sources, 22 studies on losses of income sources, and nine studies focused on losses of property. Physical costs (*n* = 75) were split between many categories, with 15 studies focused on either fatal injuries, nonfatal injuries, or transmission of zoonoses to humans. Psychological costs (*n* = 43) were mostly related to fears of wildlife-related human injuries or death (*n* = 22), followed by mental health (*n* = 9). Social costs (*n* = 56) were dominated by conflict perceptions (*n* = 42). Most wildlife crime costs (*n* = 21) were split between different forms of crime involvement, including roles as the offender (*n* = 12), intermediary (*n* = 4), or recipient of illegal items (*n* = 3). In reference to participation in conservation management (*n* = 4), three papers discussed exclusion, while only one focused on inclusion in conservation management initiatives.

## Discussion

Our systematic review found evidence that many of the costs associated with HWC are gendered but do not significantly vary across continents. We also found that many costs were skewed toward certain subcategories that have extreme consequences for humans and wildlife alike. These costs may have lasting effects on populations, with the potential to exacerbate existing conflicts.

Despite the lack of statistically significant variation in HWC across continents, it is clear, as shown in Fig. 1, that a large majority of the gendered HWC literature is in sub-Saharan Africa and East Asia. Although this may be due to regional biases related to our search terms, it may also represent a regional bias related to the gendered HWC literature generally. These contexts are important for interpreting trends and illustrate important gaps within the HWC literature.

We observed that there are many limitations to studying gender within the current HWC literature. For example, we were only able to find publications that discussed differences between a gender binary: man and woman. No other gender identities were acknowledged by researchers or claimed by those involved in the studies. Even within the binary, publications were focused on women’s HWC costs, with very few studies acknowledging men-specific HWC costs. The gender-associated costs of HWC were seldom the focus of the publications, leaving few studies that systematically assessed these costs. These limitations are important considerations when evaluating the gendered HWC literature.

### Gendered costs of human–wildlife conflict

#### Conservation management costs

Conservation management was an infrequent topic of the gendered HWC literature; only three studies—located in India, Tanzania, Brazil, and Zimbabwe—discussed exclusion from conservation management, citing barriers to entry and illiteracy as major hindrances of participation (Nabane and Matzke 1997; Rinkus et al. 2017). There are clear disparities in women’s participation in conservation in many regions of the world, including Latin America (Márquez-García et al. 2024) and southern Africa (Hunter et al. 1990). Participation in conservation management activities is often dominated by men, with women sometimes unable to participate due to the societal expectations of women’s roles outdoors and within the home (Rinkus et al. 2017).

However, there is evidence that women’s involvement in conservation can strongly benefit management initiatives. For example, women in India that were involved in ecotourism programs had more positive sentiments toward snow leopards (Alexander et al. 2022), which may in turn shape more receptive responses to wildlife management initiatives that concern the species. Yet, while women’s involvement in wildlife conservation is prevalent in Indonesia (Sheherazade et al. 2022), Mongolia (Alexander et al. 2023), and Zambia (Sommerville et al. 2022), many countries hold steadfast to traditional gender roles that prevent women from advancing in the field (Bossert et al. 2024). Including women in the conservation management sphere has the potential to add important nuance to mitigation plans, making conservation efforts more effective and efficient while also meeting the needs of those most impacted (Goldman et al. 2021; Alexander et al. 2022). For example, women’s participation may result in their increased environmental knowledge, which may aid in protecting wildlife and parks that provide natural resources to communities (Nutsugbodo and Adjei Mensah 2020).

#### Economic costs

Across the reviewed literature, economic costs related to human–wildlife conflicts were almost entirely experienced by women. These costs included losses of food and income sources and property. The main direct economic costs tied to HWC involve livestock depredation and crop destruction by wildlife searching for food and other resources on and near lands relied upon by communities. These economic losses can be devastating, especially for those living in small, rural communities that rely on subsistence to sustain their livelihoods (Khumalo and Yung 2015). In rural communities in Tanzania, women are typically responsible for their family’s agriculture, which may put them in greater contact with wildlife than men (Kaltenborn et al. 2006). People living in the buffer zones of national parks may also experience wildlife venturing closer to human settlements. This can, for example, contribute to greater instances of crop destruction, to which women may be more vulnerable (Kimaro et al. 2023). Subsistence agriculture is vital to family survival in many communities; as such, women often associate crop destruction by wildlife with famine and decreased economic gain (Costa et al. 2017). In certain cases, women may be disproportionately burdened by these losses because they are often responsible for earning additional wages outside of the home to support their families (Harvey et al. 2017) and have less access to compensation (Ogra and Badola 2008; Bhushal et al. 2024).

Women in households without men may lack the confidence or skillsets required to cope with the economic costs of human–wildlife conflict. For example, a higher rate of illiteracy in women than men in some rural communities may hinder women from applying to receive compensation for livestock lost to predation or even asking for help with the process (Ogra and Badola 2008; Ogra 2009). Women may also consider it the broader community’s responsibility to deal with such conflict costs. However, this may exacerbate limitations in the power of women’s voices. For example, (Ogra 2009) found that cooperative management in rural communities in India limits women’s voices at a governmental scale by excluding women from HWC committees. In general, the combination of high barriers to entry and women’s expectations of community support—both of which hinder women’s ability to advocate for themselves—may discourage them from attempting to solve HWC conflicts independently.

However, it is important to recognize that there are indirect economic costs that are more difficult to measure but are inequitably experienced by different genders. This includes reduced productivity from losing assets and equipment, healthcare costs associated with injuries by wildlife (Ogra 2008), or migration costs that result from no longer being able to sustain one’s family in a certain community due to recurring economic losses inflicted by wildlife (Blackie 2023). Conflict mitigation efforts may benefit from acknowledging and attempting to understand the impacts of these indirect costs on gendered responses to wildlife conflict.

We did not find clear patterns of economic cost subcategories being studied across continents. Costs associated with economic loss may be driven by a community’s proximity to wildlands or whether a government provides support for such losses and may be distributed differently through communities based on cultural values and traditions that divide the roles and expectations of men and women (Braczkowski et al. 2023). To this end, we argue that large-scale efforts to mitigate economic costs caused by wildlife conflicts are less likely to be successful when compared to small, community-based initiatives that emphasize the divergent burdens faced by people of different gender identities.

#### Physical costs

Physical costs of HWC differed significantly across gender. Violence inflicted by other people, decreased personal care and increased workload, and defense of property were more often experienced by women, whereas injury and zoonotic disease transmission costs were more often experienced by men. In instances where women are deemed responsible for economic losses, these women can face physical retaliation by their family members. Women may be beaten or even killed as punishment for shaming their families or failing to provide financial support to their husbands (Doubleday and Adams 2020). For example, women may avoid entering the forest to collect high-quality cattle fodder for fear of tiger attacks and instead feed their cattle lower quality food, leading to lower milk production (Doubleday and Adams 2020). This lower milk production may lead to women not being able to pay their dowry, and their husbands may turn to violence in response (Doubleday and Adams 2020).

The repercussions of HWC may be detrimental for women’s health. Economic loss by wildlife crop raiding can lead to decreased personal care for women, as mothers who cannot afford to feed their entire family often prioritize the wellbeing of their children over their own (Ogra 2008). In some cases, women whose husbands are jailed or killed for poaching are forced to take on the entire burden of supporting their families, which can cause immense stress (Massé et al. 2021). These costs often go largely unnoticed, as women are sometimes reluctant to share such details during interviews where men are present for fear of repercussions (Costa et al. 2017). This reluctance to share sensitive information indicates a strong presence of patriarchal dynamics in communities and may be evidenced in conservation more broadly.

Men’s household roles may lead to them experiencing different types of physical costs than women. Men are often seen as the protectors of and providers for the family; crop guarding at night and hunting are typically their responsibilities (Braga-Pereira et al. 2021; Banerjee and Sharma 2022). As such, men are often in close contact with wildlife, which can lead to injuries or death if conflicts arise (Garrote et al. 2017; Blackie 2023). Additionally, zoonotic diseases can spread through increased exposure to wildlife and from wild meat handling, which are a detriment to both human and livestock physical health (Lu et al. 2021). Responsibilities regarding hunting and preparing wild meat are often gendered, leading to differing forms of transmission across men and women (Woldehanna and Zimicki 2015; Ohemeng et al. 2017; Akem and Pemunta 2020). For example, in Hmong culture, women are typically responsible for preparing rodents, while men are more responsible for hunting and consuming them (Woldehanna and Zimicki 2015).

Similarly to economic costs, we did not find significant differences in the physical cost subcategories across continents. Culture and stigma associated with the repercussions of wildlife conflict were different across, and sometimes even within, communities and may run across gendered lines (Doubleday 2020; Corlett and Primack 2006; Doubleday and Adams 2020). Physical costs associated with injury or death by wildlife were extremely location specific, reflecting the diversity of ecosystems and wildlife people may experience across locales (Corlett and Primack 2006). These differing ecological contexts, along with diverse gender roles related to agriculture and livestock, may lead to men and women encountering different species with differing possible levels of injury severity.

#### Psychological costs

Physical and economic costs can contribute to diverse psychological traumas for those involved in human–wildlife conflicts, yet our literature review indicated that psychological costs did not vary between men and women. For those who are likely to experience close encounters with wildlife, it is common to fear injury, or even death, from physical conflicts. These fears may be exacerbated if those involved have been attacked before or have heard of others being attacked (Kaltenborn et al. 2006; Redmore et al. 2020). This is seen in the Okavango Delta, where women shift their active hours collecting firewood to avoid altercations with elephants (Redmore et al. 2020). Across various contexts, since both men and women encounter wildlife while conducting their household responsibilities, these fears may be held by both genders in many communities. For women, this anxiety may manifest during outings to collect firewood and food for livestock, which usually involves entering forested areas (Doubleday 2020), increasing their risk of encountering large carnivores like tigers and contributing to them holding negative attitudes toward the predator (Doubleday and Rubino 2022; Malviya et al. 2022; Datta et al. 2023). Further, women who cannot fulfill their household responsibilities due to this fear may also be afraid of the domestic repercussions, as punishment by spouses is not uncommon (Doubleday and Adams 2020).

Economic loss can also lead to declines in mental health. For example, in Mozambique, widows of poachers who are killed by rangers must take on the burden of providing for their families and experience immense stress on becoming the primary provider (Massé et al. 2021). In contexts for which gender is essentialized and women are caregivers, they are typically responsible for child-rearing, which can influence their outlook on wildlife. For example, women, as protectors of their household in many cultures, may be more fearful of predators like large carnivores, as they consider them to be a threat to their family’s safety (Kaltenborn et al. 2006). This fear can translate to lower tolerance for such species, including toward jaguars (*Panthera onca*) (Fort et al. 2018), sloth bears (*Melursus ursinus*) (Prajapati et al. 2021), African palm civets (*Nandinia binotata*) (Campbell 2009), and Iberian wolves (*Canis lupus signatus*) (Valente et al. 2024). This fear of wildlife’s impact on the family unit is also a result of the indirect effects of conflict, such as zoonotic disease transmission. For instance, women in Slovakia and Chile reported being more fearful of bats than men since bats may transmit disease to their children (Prokop et al. 2009; Pérez et al. 2021), demonstrating that there are multiple pathways by which wildlife conflict can influence people’s perspectives on the species involved.

Fear and disdain among women affected by HWC may exacerbate future conflicts if mitigation efforts do not directly attend to these sentiments. Khumalo and Yung (2015) detailed a scenario where the establishment of a wildlife conservancy in Namibia left women increasingly frustrated with new rules that hindered them from using their typical wildlife deterrent methods, leading not only to increased anti-wildlife sentiment, but higher rates of property destruction and economic hardship. Providing a platform for women’s concerns regarding HWC is an essential mechanism for ensuring that HWC mitigation efforts are successful. Investing in female empowerment and challenging traditional gender roles may help decrease the severity of future conflicts’ impacts on households and communities (Gore and Kahler 2012). With empowerment-driven management actions, interactions with wildlife can be made more beneficial to communities (Anthony et al. 2004).

#### Social costs and perceptions of wildlife

People’s negative attitudes toward wildlife, which comprised over 70% of the social costs recorded in our sample, were largely linked to psychological and physical costs of HWC. For example, in Uganda, women who feared altercations with African elephants while collecting firewood had more negative attitudes toward the species than men (Hill 1998). Other studies have shown that women, as caretakers of children, also disliked bats (*Chiroptera* spp.) more than men due to bats’ associations with disease risk (Prokop et al. 2009; Musila et al. 2018). A common trend that influenced these negative perceptions was a lack of exposure to potentially dangerous wildlife. In many communities, men were responsible for the defense of livestock and family, which required them to frequently spend time outdoors in places that may further expose them to wildlife (e.g., Garrote et al. 2017). In India, men, as the primary herders of livestock, report more direct experiences with wolf (*Canis lupus*) depredation than women, leading to men having more negative perceptions of the species (Bhatia et al. 2017).

While sometimes men’s increased exposure can lead to more negative attitudes toward wildlife, it more often leads to higher tolerance than women. As men spend more time interacting with wildlife, they may become more comfortable and thus be less fearful or disdainful toward species often considered threatening (Thinley et al. 2019; Pierre et al. 2023). Conversely, women whose roles confined them to the household had more negative attitudes toward leopards (*Panthera pardus*) (Dhungana et al. 2022), Sumatran elephants (*Elaphus maximus sumatrensis*) (Ardiantiono et al. 2021), and golden langurs (*Trachypithecus geei*) (Thinley et al. 2019) as compared to men. This lack of exposure to wildlife may also cause women to be more fearful and less knowledgeable about initiatives geared toward protecting wildlife, which in turn may have caused them to feel less positively toward wildlife conservation (Mir et al. 2015) and more negatively about protected area governance than men (Afriyie et al. 2022). These contradictory causes of fear of wildlife help highlight that an understanding of the contexts of and community-level responses to HWC are central for effectively managing HWC issues.

Cultural norms and expectations can be difficult to uphold for those involved in wildlife conflicts. Women who cannot pay for their dowries due to livestock depredation are shamed by their relatives and treated poorly (Doubleday and Adams 2020). Loss of family members, especially male heads of household, can lead to intense stigma against women (Datta et al. 2023). For example, ‘tiger widows’ in India, who have lost their husbands to tiger attacks, lose support systems within their communities after their husbands are killed (Chowdhury et al. 2016). Men may also experience stigma by hunting rodents, which are seen as disease-ridden (Bonwitt et al. 2016). This may be strongly intertwined with psychological costs, as the loss of community may be detrimental to people’s mental health. The connection between social and psychological costs, both of which are most often intangible, is important to consider when performing management activities or analyzing the repercussions of HWC.

Although people’s perceptions of wildlife can be based within cultural and societal norms, there is evidence that these perceptions may change because of conservation efforts such as ecotourism. In some locations, ecotourism changed women’s perceptions of snow leopards to be more positive despite depredation events of their livestock (Alexander et al. 2022). In some regions, ecotourism has allowed local women to sell goods to tourists, which is linked to women’s support for conservation of conflict-prone species, such as spotted hyenas (*Crocuta crocuta*) (e.g., Wilkinson et al. 2021). Management programs have an opportunity to decrease the gender-specific burdens associated with wildlife conflict, but women are often less receptive to wildlife conservation management programs than men due to the additional strain they experience from living near wildlife (Homewood et al. 2022). It is imperative to find management solutions that both involve women’s input and provide them with the resources and tools and support themselves and their families.

#### Wildlife crime costs

A noteworthy finding regarding wildlife crime was that it was one of two cost types *increasing* in coverage over the past decade in the literature coverage, along with psychological costs. This result parallels enhanced policy recognition to the criminal dimensions of wildlife crime at all levels—global to local (Milner-Gulland et al. 2018); it also reflects how wildlife crime is species and ecosystem agnostic and thus globally distributed. In this regard, the extant literature reflects the current tempo of policy making. Further, wildlife crime can be a type of HWC that does not embody regionality.

There are multiple roles that wildlife crime actors can assume: offender, defender, influencer, observer, person harmed, and beneficiary (Agu and Gore 2020). Although crime can be highly gendered (e.g., gendered offending and victimization, gender-based violence, sentencing and recidivism rates), our results signaled gender parity in costs experienced by wildlife crime. Wildlife crime can be both a cause and a consequence of HWC (Kurland et al. 2017). Wildlife crime can be a cause when it disrupts ecosystem function through the illegal killing of keystone species that people are less tolerant of (Shameer et al. 2024), which further fuels retaliatory attacks against species because communities continue to witness benefits from killing wildlife (Viollaz et al. 2021). Wildlife crime can be a consequence of HWC when people engage in retaliatory killing in contravention of the rule of law (Viollaz et al. 2021), including when people commodify wildlife parts for illegal trade after retaliatory killing (Gore et al. 2020), or when people instigate vigilantism when they are frustrated with or feel that the authorities are not responding to the repercussions of HWC (Woolaston et al. 2021). While the roles associated with wildlife crime may be gendered, the motivations and outcomes may be less frequently split across gendered lines, highlighting the severity of this issue and its potential to damage wildlife populations and broader ecosystems.

## Research and management implications

Gender remains an understudied but crucial aspect of human–wildlife conflict. We have shown that there are tangible costs to HWC that affect men and women in different ways and to different extents. People’s perceptions of their environment are largely dependent on whether they are lifted up or held back by the cultures and communities in which they live (Diduck 1999; Ghasemi et al. 2021). Understanding how different genders may be distinctly affected by interactions with wildlife will help allow for more responsive conservation management that makes a conscious effort to address HWC issues in a nuanced and equitable way. By doing so, managers may be more equipped to mitigate HWC, prevent HWC recurrence, and even prevent future HWC issues.

Additionally, integrating gender perspectives into human–wildlife conflict solutions aligns with global development goals; in particular, the United Nations Sustainable Development Goals (SDG) of gender equality (SDG 5) and sustainable land use (SDG 15) (Desa 2024). Creating management plans that recognize gender equity will also decrease the unequal burden of climate change on the most at-risk individuals, especially those in rural communities that are increasingly encountering wildlife along their borders (Chanamuto and Hall 2015).

Human–wildlife conflict mitigation and prevention cannot have long-term success without understanding the contexts of these interactions (Blackwell et al. 2016). It is crucial to delineate the pathways between people’s intentions and their behavior; in other words, factor in why people behave the way they do either before a human–wildlife conflict (e.g., engage in risky or risk-reducing behavior) or after a human–wildlife conflict (e.g., lethal vs nonlethal retaliation). Societal norms regarding gender differ, even at the community level. Not all communities within a country or region will necessarily respond similarly to conflicts, and within those communities, people of different genders also may not respond similarly to conflicts. Additionally, power dynamics often feed into how information is shared. People of lower status in a community or household may be fearful of retaliation if they share certain information; others who hold power in their community may also hold back information if sharing puts their position at risk. For example, in many communities, men dominate decision-making in wildlife management and conflict mitigation strategies, while women’s perspectives are underrepresented. Gender-inclusive policies and programs can support more effective and equitable HWC resolution strategies.

Oftentimes, scientists and managers may initially be successful at mitigating the proximate HWC issues in the communities they are working in, but neglect considering not only the context of the community, but also the potential cultural, societal, and gendered implications of their plans, which may exacerbate issues in the future and put vulnerable individuals at further risk (Webber et al. 2007). If managers and researchers feel it is imperative to consider the full scope of consequences from their projects and how vulnerable individuals may be impacted (Treves et al. 2009), gender must become part of the equation. Even more importantly, uplifting voices that may often be quieter, especially those of women, will enhance conservation efforts significantly by allowing different perspectives to contribute to creating more nuanced mitigation plans (Mahour 2016). Designing studies that account for context and elevate a diversity of voices and experiences is the most efficient and effective way to promote human–wildlife coexistence (Dickman 2010).

It is highly probable that people experience gendered challenges in broader conservation conflicts as well. For example, we found many studies, which we did not include in our review, regarding costs in the legal hunting and fisheries spheres. In some cases, women may use their bodies as compensation to provide food for their family, leading to increased HIV within some communities—for example, around Lake Victoria, fish-for-sex relationships are stigmatized but exceedingly common (Fiorella et al. 2015). These dynamics often lead to social, physical, and psychological costs similar to those experienced due to HWC. As another example, in some of Nigeria’s fishing villages, women are marginalized and those who cannot repay their husbands for fish to feed their families are beaten (Oloko et al. 2022). Further, men who cannot provide food for their families may experience declines in mental health and feelings of inadequacy, such as in the case of San Juan in the Philippines, where men are expected to have multiple sources of income and those that do not face scrutiny and stigma (Turgo 2015). These studies highlight that it is challenging to uncover all the hidden costs of conflicts and that the wide-ranging conservation literature should focus more attention on gendered costs.

An important limitation to our study is that all included papers were explicitly focused on the gender binary between men and women. Many studies were conducted in countries where people of genders that do not adhere to the binary are persecuted (LGBTI and Gender-Diverse Persons in Forced Displacement 2025). At the community level, there may be stigma against certain genders and people may choose not to share their gender identity for fear of retaliation (Bränström et al. 2023). Fear of persecution may hinder people from expressing their true gender identities during surveys and interviews, which then diminishes the diversity of identities that are included in the available HWC literature. This limitation adds an additional layer of difficulty to understanding the gendered impacts of HWC. To help counteract this setback, we acknowledge the opportunity for researchers to emphasize the anonymity of their subjects and encourage survey participants to share their true gender identities. We also acknowledge that researchers can address potential gender biases in their studies by being mindful of geographic context as well as the laws and gender-related norms that exist in these regions.

It is possible we have excluded studies relevant to our objectives due to the nature of our search terms. For example, our requirement of having a search term related specifically to ‘human–wildlife conflict’ in every query may have excluded some studies that use different language to describe HWC. An example of this is in the sub-Arctic, where we know outmigration of women from rural to urban areas for work can lead to increased rates of HWC for men in this region (Malik and Ford 2025). Additionally, developed nations also experience wildlife crime challenges related to biodiversity programs with attendant gender implications that are not captured in our sample. For example, men in Norway were more often involved in illegal reptile trade after a ban on keeping non-native reptiles was instated (Sollund 2022). Thus, our inclusion/exclusion criteria may be a partial cause of the regional and female-skewed bias in the dataset. Similarly, excluding ‘coexistence’ as one of our search terms may have excluded some relevant studies but likely would have also yielded many studies outside the scope of our review. Although including a larger set of search terms that could capture broader depth of studies related to HWC may have been useful for our analysis (e.g., outmigration), it may have also led to a high number of irrelevant studies as well. Our review is focused on the causes of conflicts rather than solutions for conflicts, which leaves room for additional discourse on the outcomes of existing management initiatives and potential solutions to HWC. While understanding the causes of conflict is crucial to mitigating them, recognizing feasible solutions and the contexts in which they have been successful is important for developing effective management plans as well.

Communities hold different values, traditions, and roles for their members and place differing importance on wildlife; these factors determine whether they respond positively or negatively to management and policy. We observe a clear opportunity for robust empirical research on the intersection between gender and human–wildlife conflict, conducted in meaningful consultation with communities. Future studies could examine the gendered cause and effect of conflicts, which will increase the effectiveness and equity of management initiatives.

## Supplementary Information

Below is the link to the electronic supplementary material.

Supplementary file1 (PDF 242 KB)

## Data Availability

Data used in this manuscript are available online at 10.5281/zenodo.16050318.
