# Supplementary material for: The gendered costs of human–wildlife conflict: A global systematic review
Source: Ambio. 2025 Nov 7;55(6):1165–80. doi: 10.1007/s13280-025-02300-y (PMC13125431; doi:10.1007/s13280-025-02300-y)
Supplement: Supplementary file 1 — Supplementary file1 (PDF 242 KB) [file 13280_2025_2300_MOESM1_ESM.pdf]

Supplementary Information: This Supplementary Information has not been peer-reviewed.

Title: The gendered costs of human-wildlife conflict: A global systematic review.

Fig. S1 PRISMA flow diagram of record screening for the Web of Science database search

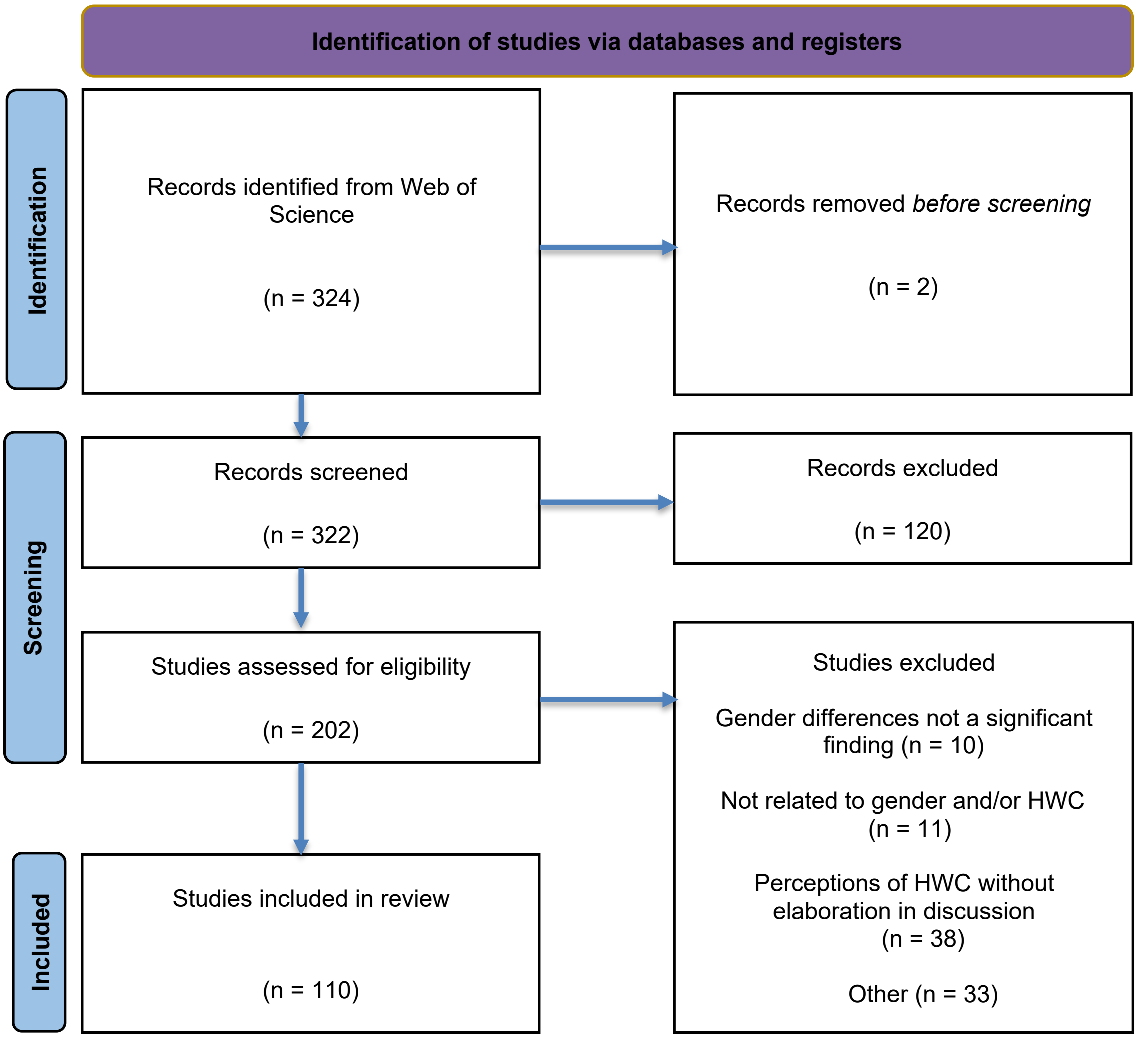

Fig. S2 PRISMA flow diagram of record screening from the backwards search of publications included in the review

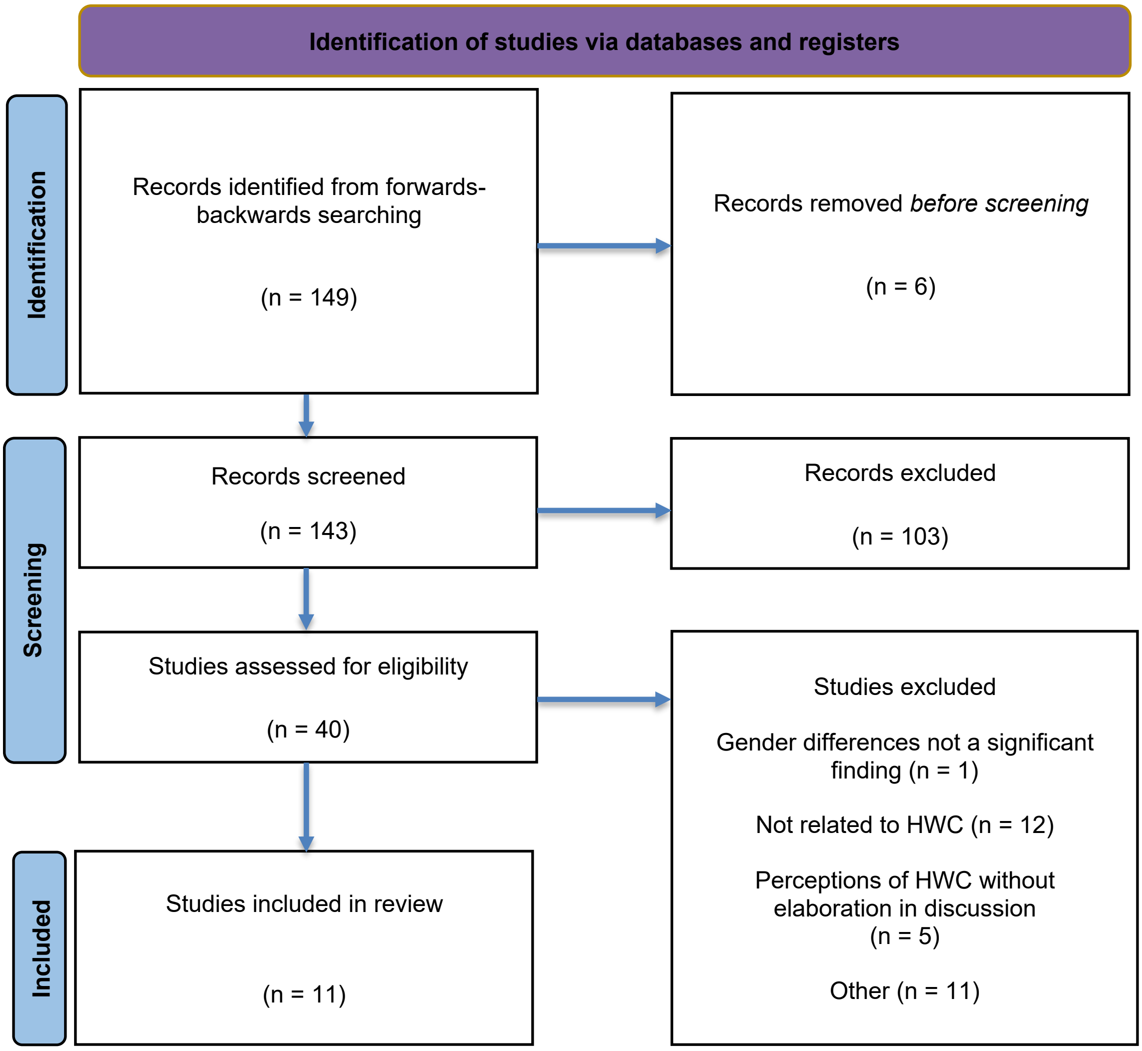

Source: Page MJ, et al. BMJ 2021;372:n71. doi: 10.1136/bmj.n71.

This work is licensed under CC BY 4.0. To view a copy of this license, visit https://creativecommons.org/licenses/by/4.0/
